# Regulating bile acids signaling for NAFLD: molecular insights and novel therapeutic interventions

**DOI:** 10.3389/fmicb.2024.1341938

**Published:** 2024-06-03

**Authors:** Meilin Wei, Wei Tu, Genhua Huang

**Affiliations:** ^1^Department of Endocrinology and Metabolism, The Second Affiliated Hospital of Nanchang University, Nanchang, China; ^2^Department of Obstetrics and Gynecology, The Second Affiliated Hospital of Nanchang University, Nanchang, China

**Keywords:** bile acids, gut microbiota, NAFLD, TGR5, FXR, gut liver axis

## Abstract

Nonalcoholic fatty liver disease (NAFLD) emerges as the most predominant cause of liver disease, tightly linked to metabolic dysfunction. Bile acids (BAs), initially synthesized from cholesterol in the liver, undergo further metabolism by gut bacteria. Increasingly acknowledged as critical modulators of metabolic processes, BAs have been implicated as important signaling molecules. In this review, we will focus on the mechanism of BAs signaling involved in glucose homeostasis, lipid metabolism, energy expenditure, and immune regulation and summarize their roles in the pathogenesis of NAFLD. Furthermore, gut microbiota dysbiosis plays a key role in the development of NAFLD, and the interactions between BAs and intestinal microbiota is elucidated. In addition, we also discuss potential therapeutic strategies for NAFLD, including drugs targeting BA receptors, modulation of intestinal microbiota, and metabolic surgery.

## 1 Introduction

As the global incidence of obesity and its associated metabolic syndrome escalates, nonalcoholic fatty liver disease (NAFLD) has emerged as a primary contributor to chronic liver conditions and progressive liver fibrosis. Its global prevalence is estimated to be around 25% (Powell et al., 2021). NAFLD encompasses a continuum of pathological changes, ranging from simple steatosis (non-alcoholic fatty liver, NAFL) to nonalcoholic steatohepatitis (NASH), then progressing toward fibrosis and ultimately leading to hepatocellular carcinoma and liver failure. Approximately 5–25% of individuals with nonalcoholic steatohepatitis progress to severe liver fibrosis (Castera et al., 2019).

The development of NAFLD is inherently intricate and governed by numerous factors. Originally, the pathogenesis of NAFLD was described by the “two-hit” hypothesis: the “first hit” being lipid accumulation in the liver, which predisposes the organ to further damage, and the “second hit” comprising factors like lipotoxicity, mitochondrial damage, oxidative stress, and hepatic inflammation, which facilitate the progression from NAFL to liver fibrosis. More recently, the understanding has shifted to the “multiple-hit” hypothesis. This theory includes various detrimental factors, including insulin resistance, inflammatory mediators, dietary factors, gut microbiota imbalances, and genetic and epigenetic variations (Kumar et al., 2021). However, the exact pathogenesis of NAFLD is largely unknown, and no FDA-approved drug to treat NAFLD is currently available.

Recent studies have increasingly focused on the role of bile acids (BAs) in the pathogenesis of NAFLD due to their origin from hepatic cholesterol (Arab et al., 2017). Dysregulation in BA metabolism in NAFLD patients heightens the risk of liver damage. Research has demonstrated marked disparities in both the serum levels and composition of BAs between high-fat mouse models and NAFLD patients, and those observed in a normal control group (Jiao et al., 2018). Furthermore, elevated levels of circulating BAs have been detected in diet-induced NASH mice, correlating closely with the severity of liver fibrosis (Suga et al., 2019). In patients with NASH, enhanced BA synthesis compared to healthy individuals has been noted, and serum BA levels are predictive of liver fibrosis severity (Shlomai et al., 2013; Mouzaki et al., 2016). In addition to assisting the absorption of dietary lipids and vitamins, BAs are crucial signaling molecules that regulate lipid and glucose metabolism and modulate inflammation in various tissues. The imbalance of gut microbiota is intricately linked to NAFLD progression (Henao-Mejia et al., 2012; Aron-Wisnewsky et al., 2020). BAs, as one class of metabolites of intestinal microbiota, are frequently associated with metabolic diseases including obesity, diabetes and NAFLD. It has been demonstrated that administration of beneficial bacteria could improve NAFLD (Kolodziejczyk et al., 2019). On the other hand, metabolites derived from microbiota can also reduce NAFLD severity by mediating the beneficial effects of intestinal bacteria.

In this review, we synthesize and analyze the mechanisms through which BA signaling influences NAFLD pathogenesis, focusing particularly on the interactions between BAs and gut microbiota, as well as identifying potential therapeutic targets for the treatment of NAFLD.

## 2 BA synthesis and regulation

### 2.1 BA synthesis

**Figure 1:**
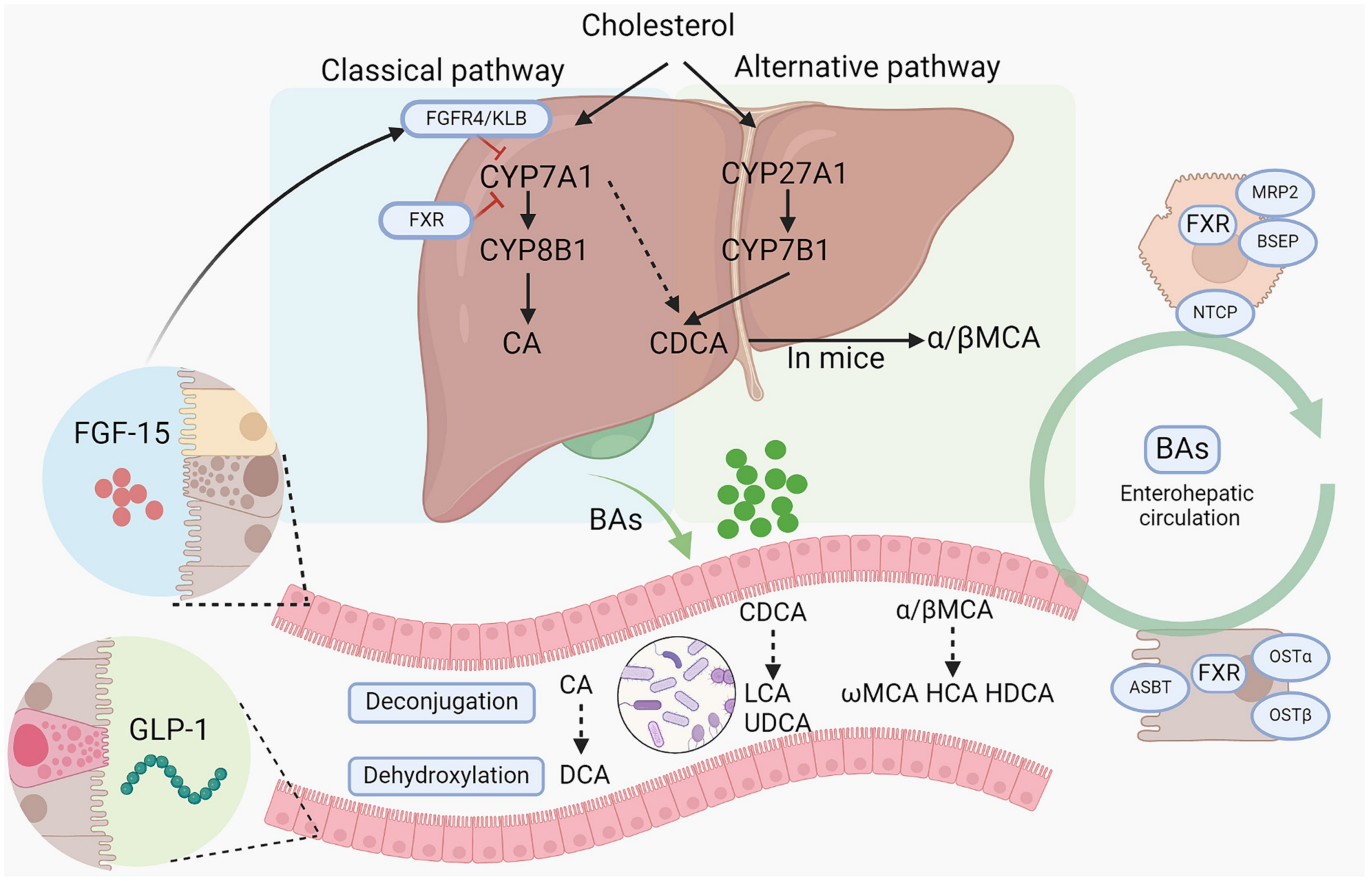
Bile acid synthesis and metabolism by gut microbiota. Bile acids are synthesized from cholesterol via classical pathway and alternative pathway. CYP7A1, CYP27A1, CYP8B1, and CYP7B1 are the main enzymes responsible for primary BA synthesis. Then conjugated BAs are released into intestine and further metabolized by gut microbiota into secondary BAs via a series of action including deconjugation, dihydroxylation, and epimerization. And primary bile acids and secondary bile acids can cooperate to stimulate the intestinal epithelium to secrete FGF15 and GLP-1 to regulate BA synthesis in turn and alter host metabolism. BAs, bile acids; CYP7A1, Cholesterol 7-alpha hydroxylase; CYP8B1, sterol 12α-hydroxylase; CYP27A1, cholesterol 7a-hydroxylase; CYP8B1, sterol-12α-hydroxylase; CA, cholic acid; CDCA, chenodeoxycholic acid; DCA, deoxycholic acid; LCA, lithocholic acid; HDCA, hyodeoxycholic acid; HCA, hyocholic acid; LCA, lithocholic acid; FGF15/19, fibroblast growth factor 15/19; MCAs/ α/β-MCA, muricholic acids/α/β-muricholic acid; UDCA, ursodeoxycholic acid; GLP-1, glucagon-like peptide-1; ASBT, apical sodium-dependent BA transporter; FXR, farnesoid X receptor; OSTα/β, organic solute transporter subunit α and β; NTCP, sodium dependent taurocholate co-transporting polypeptide; BSEP, bile salt export pump; KLB: β-Klotho.

BAs are synthesized predominantly from cholesterol in the liver through two primary pathways: the classical pathway and the alternative pathway, involving at least 17 enzymes (Figure 1). The classical pathway, initiated by the rate-limiting enzyme cholesterol 7a-hydroxylase (CYP7A1) and further regulated by sterol-12α-hydroxylase (CYP8B1), generates the majority of the BA pool. In contrast, the alternative pathway is chiefly controlled by sterol 27α-hydroxylase (CYP27A1) and sterol 7α-hydroxylase (CYP7B1), producing chenodeoxycholic acid (CDCA) and cholic acid (CA) in humans, and predominantly beta-muricholic acid (β-MCA) in rodents (Wahlström et al., 2016). CYP8B1 plays a crucial role in synthesizing CA, thus determining the CA to CDCA ratio (Bertaggia et al., 2017). Within hepatocytes, BAs are conjugated primarily with glycine in humans and almost exclusively with taurine in mice, forming conjugated bile acids that are expelled into the bile through the bile salt export pump (BSEP) and multidrug resistance-associated protein 2 (Falany et al., 1994). Released into the duodenum, primary bile acids are transformed into secondary bile acids like deoxycholic acid (DCA) and lithocholic acid (LCA) by intestinal bacteria via deconjugation and dehydroxylation. Subsequently, over 95% of the BAs are reabsorbed into the portal vein by the apical sodium dependent bile acid transporter (ASBT) at the terminal ileum and returned to the liver, constituting the enterohepatic circulation, while the remainder is excreted in the feces (de Aguiar Vallim et al., 2013). BAs re-enter the liver from the bloodstream via the Na^+^-taurocholate cotransport polypeptide (NTCP). BA homeostasis is a critical physiological process involving the synthesis, metabolism, and recycling of bile acids, which are essential for lipid digestion and nutrient absorption. The regulation of BA levels is finely tuned by feedback mechanisms primarily mediated by BA receptors, which inhibits bile acid synthesis in the liver when intrahepatic or intestinal bile acid levels are high. Disturbances in bile acid homeostasis are associated with liver diseases such as cholestasis and NAFLD, as well as systemic disorders including obesity and diabetes (Wahlström et al., 2016). For instance, ASBT inhibitor blocks the reabsorption of BAs in the terminal ileum, thereby increasing BA excretion in feces and improving NAFLD induced by high fat diet (Rao et al., 2016).

### 2.2 BA receptors

The primary BA receptors are the farnesoid X receptor (FXR), or NR1H4, and the G protein-coupled bile acid receptor (TGR5). FXR is predominantly expressed the liver, intestines, white adipose tissue, adrenal glands, kidneys, and immune cells (Lefebvre et al., 2009). The binding affinity of BAs to FXR follows the order: CDCA > DCA > CA > LCA. Conversely, Tα-MCA, Tβ-MCA, and possibly UDCA act as antagonists (Sayin et al., 2013). TGR5 is expressed in brown adipose tissue (BAT), enteroendocrine L cells, white adipose tissue (WAT), gallbladder, skeletal muscle, islet α and β cells, immune cells, astrocytes and neurons, and is activated by BAs with varying efficacies (LCA > DCA > CDCA > CA) (Guo et al., 2016). Additional receptors involved in BA signaling include the vitamin D receptor (VDR), liver X receptor (LXR), pregnane X receptor (PXR), and sphingosine-1-phosphate receptor 2 (SIPR2) (Supplementary Table S1) (Schaap et al., 2014). BAs can exert a wide range of regulatory effects through these receptors, including glucose and lipid metabolism, BA homeostasis and energy expenditure, immune and inflammatory responses, and improving insulin sensitivity (Thomas et al., 2008).

### 2.3 BA synthesis regulation

By regulating FXR receptors in the liver and intestine, BAs can achieve self-regulation and control the transport of BAs, maintaining BA balance (Mencarelli and Fiorucci, 2010). FXR knockout mice show increased CYP7A1 expression and hepatic BA synthesis, suggesting that FXR primarily mediates the inhibitory effects of BA synthesis via CYP7A1 (Sinal et al., 2000). This suppression primarily occurs through the interaction of the small heterodimer partner (SHP) with liver receptor homolog-1 (LRH-1), curtailing CYP7A1 gene expression (Goodwin et al., 2000), a mechanism critical for averting excessive BA production and resultant liver damage. Furthermore, intestinal FXR activation by BAs at the ileum’s terminal segment prompts fibroblast growth factor 15 (FGF15), analogous to human FGF19, to modulate bile acid synthesis in the liver. Upon FGF15/FGF19 is secreted, it binds to the hepatic fibroblast growth factor receptor 4 (FGFR4)/beta-klotho heterodimer complex, triggering JNK1/2 and ERK1/2 signaling cascades, leading to the inhibition of CYP7A1 expression (Potthoff et al., 2012). Experimental data from tissue-specific FXR knockout mice indicate intestinal FXR exerts a stronger inhibitory impact on CYP7A1 compared to hepatic FXR, which more significantly influences CYP8B1 expression and thus reduces cholic acid production (Kim et al., 2007; Kong et al., 2012). The gut-liver axis, delineating the complex interactions between gut microbiota and the liver, is closely linked to NAFLD (Song and Zhang, 2022). Microbial metabolites and other intestinal signaling molecules could reach the liver via portal and systematic circulation, regulating BAs synthesis and transport. Of note, intestinal FXR-FGF15/19 plays a critical role in the crosstalk of gut microbiota and BAs. FGF15/19 has displayed substantial protection against hepatic steatosis caused by high-fat diets (Sciarrillo et al., 2021). Continued clinical investigations are imperative to explore the therapeutic potentials of FGF19-based chimeric molecules for NAFLD therapy.

## 3 BA signaling in metabolism

**Figure 2:**
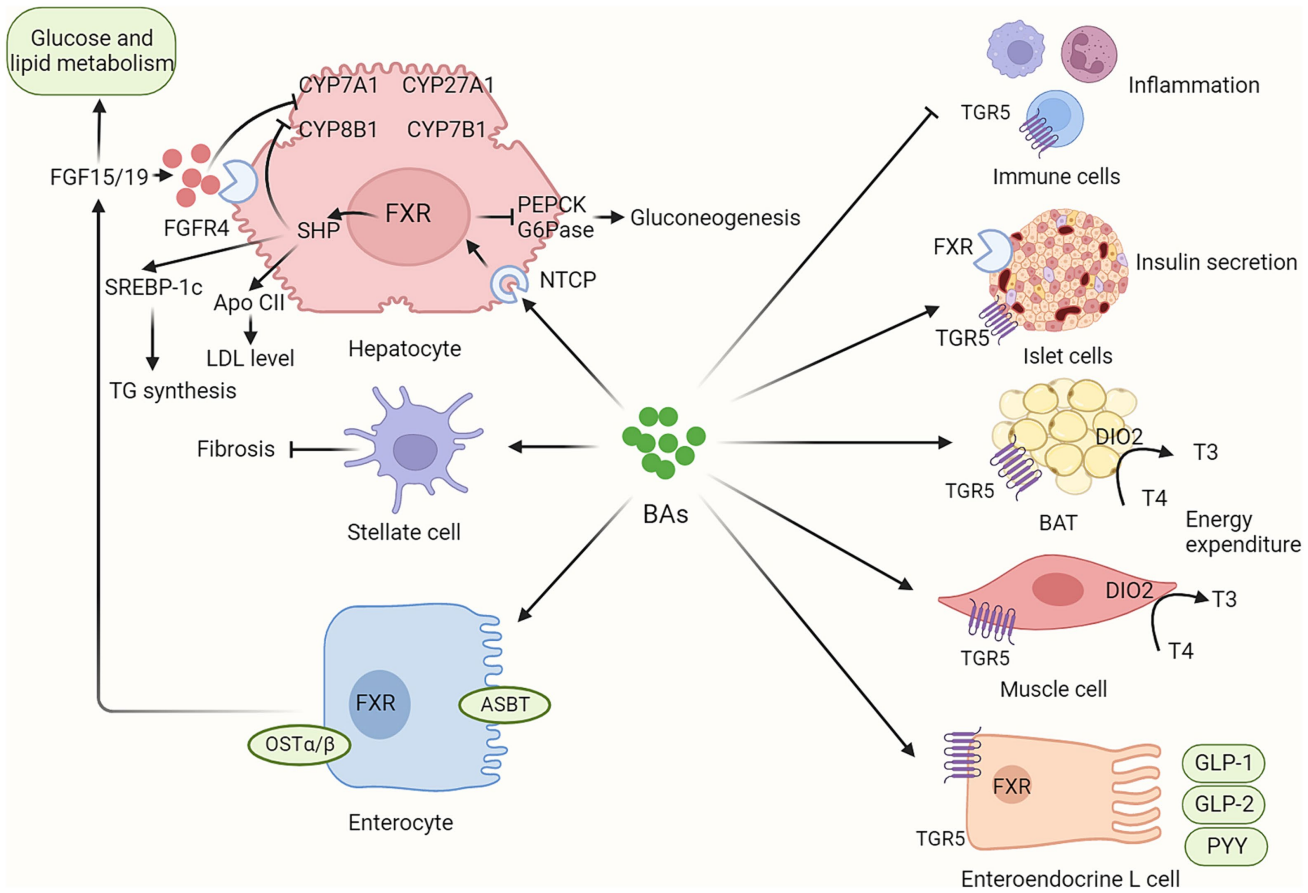
BAs contribute to host metabolism in various organs via their receptors. BAs synthesis is not only regulated by hepatic FXR/SHP signaling but also by intestinal FXR/FGF15/19 signaling. In addition, circulating FGF15/19, as metabolic hormones, could improve glucose and lipid metabolism. BAs contribute to host metabolism in multiple organs via their FXR and TGR5 receptors, including enhancing insulin secretion in pancreatic islet cells, promoting energy expenditure in brown adipose tissue and muscle tissue, inducing incretin release in enteroendocrine L cells, and regulating inflammation response in immune cells. BAs, bile acids; ASBT, apical sodium-dependent bile acid transporter; BAT, brown adipose tissue; CYP7A1, Cholesterol 7-alpha hydroxylase; CYP8B1, sterol 12α-hydroxylase; CYP27A1, cholesterol 7a-hydroxylase; CYP8B1, sterol-12α-hydroxylase; DIO2, Type II iodothyronine deionidinase; FGF15/19, fibroblast growth factor 15/19; FGFR4, fibroblast growth factor receptor 4; FXR, farnesoid X receptor; G6Pase, glucose 6-phosphatase; GLP-1, glucagon-like peptide-1; GLP-2, glucagon-like peptide-2; NTCP, sodium dependent taurocholate co-transporting polypeptide; PEPCK, phosphoenolpyruvate carboxykinase; OSTα/β, organic solute transporter subunit α and β; PYY, peptide YY; TGR5, G protein-coupled bile acid receptor.

BAs play a critical role in various metabolic processes via their receptors, mainly FXR and TGR (Figure 2). Dysregulated BAs signaling contributes to the initiation and progression of NAFLD.

### 3.1 BA signaling in glucose metabolism

Insulin resistance is a primary characteristic of NAFLD, integral to both its development and progression. BAs are released into the digestive tract after a meal. Therefore, as postprandial messengers, BAs regulate glucose metabolism by activating various receptors. Studies using whole-body FXR knockout mice have shown a reduction in insulin sensitivity, whereas administration of the FXR agonist GW4064 significantly improves insulin resistance and glucose regulation in ob/ob mice (Cariou et al., 2006). Additionally, hepatic gluconeogenesis, vital for maintaining glucose levels, is attenuated by FXR activation, which reduces the expression of critical enzymes such as glucose 6-phosphatase (G6Pase) and phosphoenolpyruvate carboxykinase (PEPCK) (Ma et al., 2006). Moreover, BAs enhance insulin synthesis and secretion by stimulating the release of glucagon-like peptide 1 (GLP-1) via TGR5 activation. This mechanism not only protects pancreatic β-cells from apoptosis but also encourages their proliferation (Zheng et al., 2021). Furthermore, recent research indicates that TGR5, expressed in pancreatic α and β-cells, promotes insulin secretion and stabilizes glucose levels (Kumar et al., 2012, 2016). Activation of LXR increases the expression of the GLUT4 in adipose and muscular tissues, thereby improving glucose uptake (Baranowski et al., 2014). In addition, BAs interact with FXR in ileal enterocytes to trigger FGF15/19 signaling, enhancing glucose control (Kliewer and Mangelsdorf, 2015).

### 3.2 BA signaling in lipid metabolism

NAFLD is marked by significant lipid accumulation in the liver and BAs exert a profound effect on lipid metabolism mainly via FXR signaling. Notably, FXR-deficient (FXR^−/−^) mice exhibit marked elevations in hepatic and plasma cholesterol and triglycerides. Conversely, FXR agonists reduce these lipid parameters in db/db mice, yet show no efficacy in FXR deficient mice (Cariou et al., 2006). In NAFLD patients, FXR expression is reduced, which correlates with an increase in sterol regulatory element binding protein-1c (SREBP-1c) (Yang et al., 2010). BAs act on hepatic FXR and induce SHP expression, thereby inhibiting SREBP-1c and reducing fatty acid synthesis (Watanabe et al., 2004). FXR further inhibits the expression of the apolipoprotein (apo) B gene, decreasing very low-density lipoprotein (VLDL) secretion (Hirokane et al., 2004). Additionally, FXR boosts lipoprotein lipase activity by upregulating the expression of apolipoprotein C-II (apo-CII), a stimulator of lipoprotein lipase, while decreasing apolipoprotein C-III (apo-CIII) expression, a lipoprotein lipase inhibitor (Chavez-Talavera et al., 2017). The hydrophobicity and conjugation state of the BAs are critical for regulating intestinal cholesterol and lipid absorption. Thus, CA absence in CYP8B1 knock-out mice prevents hepatic steatosis induced by a high-fat diet (Bertaggia et al., 2017).

### 3.3 BA signaling in energy expenditure

Currently, obesity is believed to be the result of a disruption in energy metabolism, where energy intake exceeds energy expenditure. Thus, enhancing energy expenditure presents a good approach for NAFLD therapy. Activation of TGR5 by BAs elevates brown fat tissue energy expenditure, improve insulin resistance, and prevent obesity (Zietak and Kozak, 2016). It also results in elevated cAMP levels, which in turn activates type 2 iodothyronine deiodinase (D2). This activation enhances the conversion of thyroxine (T4) into the more active form, triiodothyronine (T3). Subsequently, T3 boosts uncoupling protein 1 (UCP-1) expression, further enhancing energy expenditure. Therefore, TGR5 signaling mediated by BAs plays a pivotal role in maintaining energy homeostasis (Watanabe et al., 2006). In contrast, TGR5-deficient mice exhibit heightened obesity susceptibility under a high-fat diet compared to control group (Maruyama et al., 2006). A clinical trial involving 12 healthy women who received chenodeoxycholic acid (CDCA) treatment for 2 days revealed significant increases in brown fat energy expenditure. *In vitro*-cultured human brown fat cells exposed to CDCA also showed an increase in UCP1 expression levels (Broeders et al., 2015).

Therefore, regulating bile acids to enhance the TGR5 signaling pathway and increase brown fat energy expenditure could become an important target for NAFLD. However, despite its positive impact on metabolic health, therapeutic targeting of TGR5 signaling is hindered by the potential for TGR5-mediated gallstone formation and gallbladder filling. Therefore, TGR5-selective agonists, such as INT-777, RDX8940, show promising effects in improving energy metabolism, reducing inflammation and stimulating energy expenditure (Pellicciari et al., 2009; Finn et al., 2019).

### 3.4 BA signaling in inflammation

Recently, the association between bile acids and immune regulation is an emerging and increasingly studied field in biomedical research. Activation of the innate immune system is pivotal in initiating hepatic inflammation, while persistent low-grade inflammation is crucial to the development of NAFLD and liver fibrosis. Recent studies highlight the significance of BA signaling in regulating hepatic inflammation. TGR5 and FXR are localized in various immune cell types, such as monocytes and macrophages, as well as dendritic cells (Fiorucci et al., 2021). Research has shown that activation of TGR5 decreases cytokine production in monocytes and macrophages and exerts potent anti-inflammatory effects (Perino and Schoonjans, 2015). Moreover, TGR5 activation protects against inflammation induced by lipopolysaccharide (LPS) by suppressing the production of proinflammatory cytokines mediated by NF-κB pathway (Wang et al., 2011). On the contrary, TGR5 deficiency promotes NLRP3 inflammasome and M1 macrophage polarization (Ma et al., 2021) and TGR5(^−/−^) mice show increased liver inflammation (Wang et al., 2011). M1 macrophages have the capability to secrete pro-inflammatory cytokines, including IL-1β, IL-6, TNF-α. In addition, FXR agonist can also orchestrate the immunological activities of macrophages and monocytes to improve NAFLD (McMahan et al., 2013). FXR activation decreased the mRNA levels of inflammatory genes (IL-1β, IL-6 and TNF-α) induced by LPS treatment *in vitro* (Xiong et al., 2017). *In vivo*, activation of the intestinal FXR signaling inhibits inflammation and helps maintain the integrity of the intestinal barrier in inflammatory bowel disease (Gadaleta et al., 2011). BAs homeostasis also plays an important role in inflammation regulation. Overexpression of CYP7A1 has been shown to protect the liver from inflammatory infiltration and alleviate hepatic fibrosis in FXR dependent manner. Recently, 3-oxoLCA and isoallo-LCA, which are derived from LCA, have been identified as T_H17_ and Treg cell regulators to control their differentiation, suggesting BAs regulate host immune response (Hang et al., 2019). Therefore, regulating BA signaling has emerged as a promising strategy in treating NAFLD by attenuating hepatic inflammation. Recent therapeutic advances involve the use of FXR agonists, which have shown efficacy in reducing hepatic steatosis and inflammation (Zhang et al., 2009).

## 4 Gut microbiota and BAs

The gut microbiota is regarded as a metabolic “organ” that produces numerous metabolites to regulate host metabolism. The interaction between gut microbiota and BAs is a significant area of research because it underscores a complex, bidirectional relationship where not only does the gut microbiota influence BAs profiles, but BAs also affect the composition and function of the gut microbiota.

### 4.1 Microbial regulation of BAs

Microbial metabolism of BAs by gut microbiota not only increase the diversity of BAs but also promote the hydrophobicity of the BA pool. The BA pool and composition in germ-free (GF) mice exhibit significant differences compared to those in conventionally raised mice (Sayin et al., 2013), emphasizing the vital role of gut microbiota in bile acid regulation. Bile acid deconjugation is the first step of metabolism by bacteria with bile salt hydrolase (BSH) activity, which is present in strains of lactobacilli, bifidobacteria, Bacteroides, and Clostridium. Deconjugated primary BAs are further metabolized through the 7-dehydroxylation into secondary bile acid. The potent endogenous agonists of TGR5 are LCA and DCA, which are metabolically derived from CDCA and CA, respectively. This transformation is primarily governed by intestinal bacteria, including Clostridium (such as *C. scindens* and *C. sordellii*), Bacteroides (such as *B. fragilis*), and Eubacterium (such as *E. lentum*). At lower concentrations, these metabolites positively influence glucose and lipid metabolism. However, when present at higher concentrations, they exert negative effects on host health. For example, fecal DCA levels are significantly elevated in patients with NAFLD compared to those in the healthy control group (Kasai et al., 2022), thereby promoting obesity-associated hepatocellular carcinoma (HCC) by causing DNA damage (Yoshimoto et al., 2013). Therefore, maintaining the homeostasis of intestinal microflora and its metabolites is essential. For example, GF mice has increased bile acid pool and TβMCA level, which serves as an antagonist to intestinal FXR signaling, thus inducing FGF15/19 secretion to inhibit hepatic BA synthesis. Some probiotics can regulate the metabolism and synthesis of BAs, thereby influencing the body’s metabolism and the progression of diseases. Recent research demonstrated that *lactobacillus rhamnosus GG* increased ileum FGF15 and subsequently reduced BA synthesis, which attenuated liver inflammation and prevents liver fibrosis (Liu et al., 2020). Levels of *Parabacteroides distasonis* are reportedly lower in individuals with NAFLD, and this bacterium can mitigate obesity and metabolic dysfunctions by modulating the production of UDCA and LCA (Wang et al., 2019).

### 4.2 BAs regulating gut microbiota

Gut microbiota plays a crucial role in maintaining BA homeostasis through via bioconversion of BA and enhancement of FGF15/FGF19 signaling. On the other hand, BAs, serving as the detergent molecules in intestine, significantly shape the composition of the intestinal microflora. BAs exert antimicrobial properties, affecting the growth and survival of different strains. Zheng et al. showed that mice fed with BAs under a normal diet condition exhibited obese phenotype and similar gut microbial composition in HFD mice (Zheng et al., 2017). A recent study showed that obeticholic acid (OCA), an analog of CDCA, inhibited endogenous BA synthesis and led an increased proportion of Firmicutes in the small intestine (Friedman et al., 2018). Gut microbiota modulates NAFLD in part via regulating intestinal FXR signaling. In obese mice induced by HFD, glycine-β-muricholic acid (Gly-MCA) administration reduced the ratio of *Firmicutes* to *Bacteroidetes,* which improved insulin resistance and ameliorated obesity related metabolic dysfunction (Zhang et al., 2016). Although serum BAs concentrations are elevated in individuals with NAFLD, the proportion of the FXR antagonistic DCA has increased, whereas the levels of the FXR agonistic CDCA have decreased (Jiao et al., 2018). Therefore, FXR signaling can modulate the gut microbial composition and regulate hepatic metabolism.

## 5 Targeting BA metabolism for NAFLD therapy

**Figure 3:**
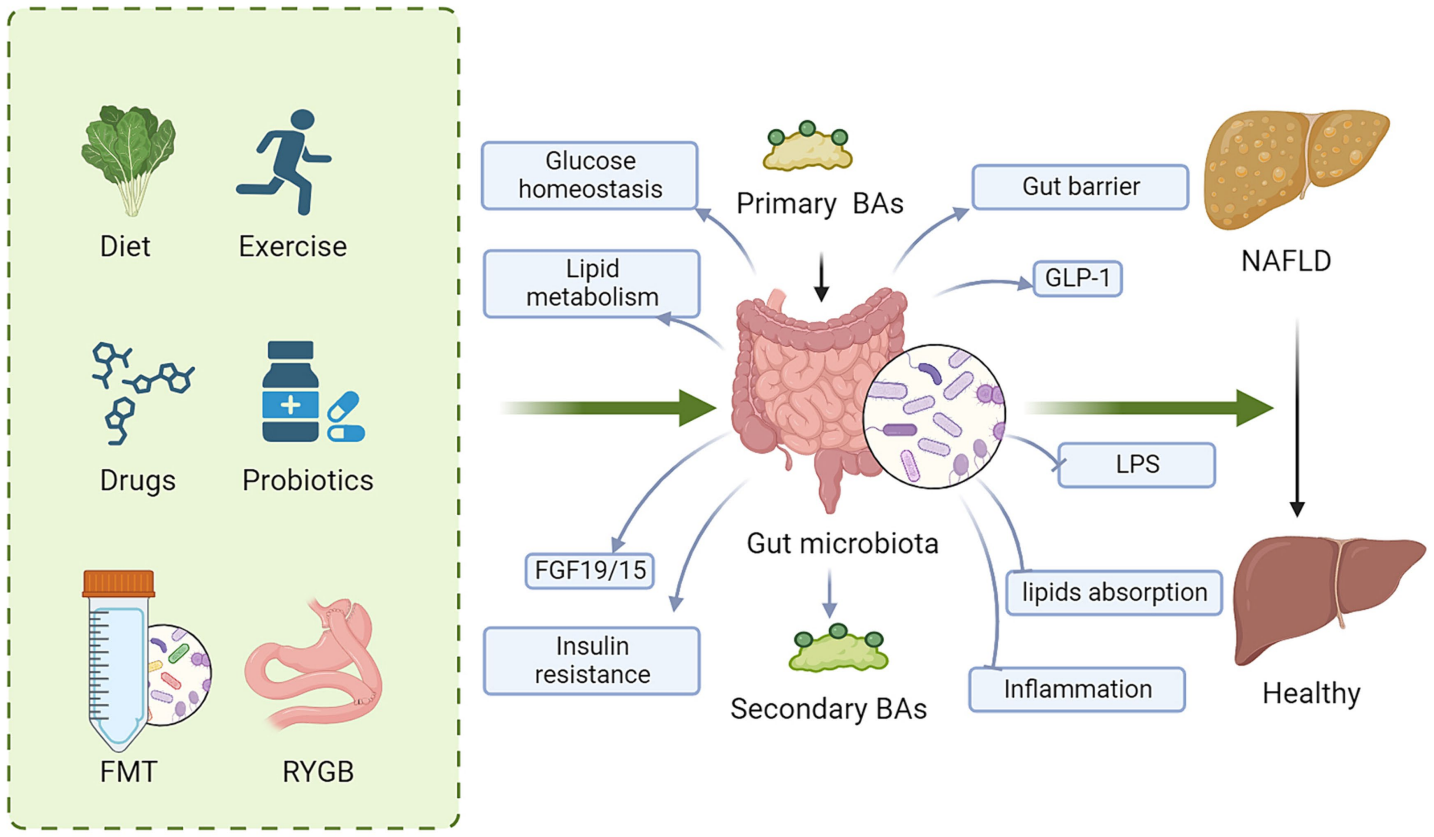
Regulating BA signaling is a promising therapeutic target for NAFLD. These approaches to regulate BA signaling for NAFLD therapy include diet, exercise, drugs, probiotics, FMT and metabolic surgery. Mechanically, targeting BAs and gut microbiota improves glucose and lipid metabolism, gut barrier, insulin resistance and GLP-1 secretion, while decreases lipid absorption, LPS, and inflammation, ameliorating hepatic steatosis. BAs, bile acids; FMT, fecal microbiota transplantation; FGF15/19, fibroblast growth factor 15/19; LPS, lipopolysaccharide; GLP-1, glucagon-like peptide-1; NAFLD, nonalcoholic fatty liver disease.

Targeting BA metabolism has become a promising therapeutic strategy for NAFLD due to the significant impact of BA on host metabolism, energy expenditure, inflammation, and the composition of the gut microbiota. Significant efforts have been made to explore approaches that modify BAs signaling. These approaches include agonist for BA receptor, regulating gut microbiota and metabolic surgery (Figure 3).

### 5.1 Pharmacotherapy for NAFLD by targeting BA

Traditional pharmacotherapies for NAFLD, including PPAR agonists, insulin sensitizers, and antioxidants, have been extensively researched; however, their effectiveness varies among individuals and is generally limited. It is well-established that activation of BA receptors exhibits metabolic benefits in NAFLD. Emerging evidence strongly suggests that BA receptors are promising therapeutic targets. Currently, a wide range of FXR agonists are under investigation in clinical trials for NAFLD. OCA, a highly potent FXR agonist and a semi-synthetic derivative of CDCA, has been approved by the FDA for treating patients with PBC who are unresponsive to UDCA (Trauner et al., 2019). Meanwhile, OCA has significantly enhanced the histological features associated with NASH. However, LDL cholesterol was increased in the early stage of OCA treatment (Younossi et al., 2019). In addition to common side effects such as pruritus, long term OCA administration may increase the risk of cardiovascular disease. Several other FXR agonists, including EDP305, tropifexor and cilofexor have already undergone phase II clinical trials, demonstrating the potential to emerge as novel treatments for NAFLD (Panzitt et al., 2022). Fexaramine, a non-absorbable FXR agonist that remains in the intestine, has shown potential in reducing diet-induced obesity and improving insulin sensitivity (Fang et al., 2015). The TGR5-specific agonist INT-777, derived from CA, alleviated liver fat accumulation and improved insulin sensitivity in obese mice (Thomas et al., 2009). Recently, a novel TGR5 agonist RDX8940, has been reported to improve hepatic steatosis in western diet-fed mice and enhance the secretion of gastrointestinal hormones such as GLP-1, GLP-2, and peptide YY (Finn et al., 2019). BA receptor dual agonists target multiple receptors involved in BA signaling, which play crucial roles in metabolic regulation, inflammation control, and liver protection. Research indicates that INT-767, an agonist for both FXR and TGR5, can improve metabolic control, reduce liver fibrosis, and decrease inflammation in animal models of liver disease (Comeglio et al., 2018; Jadhav et al., 2018; Roth et al., 2018). This makes it a promising candidate for the treatment of NAFLD and NASH. Sevelamer, as a BA sequestrant, has been shown to alleviate hepatic inflammation, lipid deposition, and fibrosis by targeting LPS signaling and inducing BAs excretion (Tsuji et al., 2020). Drugs that inhibit ASBT reduce the reabsorption of ileal BAs, enhancing their synthesis from cholesterol in liver and excretion in feces through suppression of FGF15/19. ASBT inhibitors, such as IMB17-15, volixibat and elobixibat, currently being investigated for their potential to treat NAFLD and NASH (Ge et al., 2019; Jansen, 2021). Additional clinical trials and long-term follow-ups are necessary to fully understand their impact on host health.

### 5.2 Regulating gut microbiota for NAFLD

There are several strategies to regulate the intestinal microbiota and further improve NAFLD, including the diet and exercise, the administration of probiotics, prebiotics, and fecal microbiota transplantation (FMT). For example, high fat diet caused increased DCA levels, accompanied by alteration of gut microbiota (Lin et al., 2019). In contrast, calorie restriction significantly altered microbiota and decreased the levels of non-12α-hydroxylated BAs (Li et al., 2022). Exercise could also regulate intestinal microbiota and induce metabolic improvements by modifying circulating BAs (Aoi et al., 2023). The molecular mechanism underlying metabolic improvement by exercise may involve FXR-FGF15 signaling (Qiu et al., 2021). Increasing evidence supports that various probiotic bacteria have promising effects on NAFLD, including the genera such as *Bifidobacteria* and *Lactobacillus*, along with others like *Saccharomyces boulardii* and *Streptococcus thermophilus*. Zhao et al. showed that administration of *Lactobacillus plantarum* alleviated the severity of NAFLD in HFD induced mice (Zhao et al., 2020)*. Lactobacillus gasseri* ameliorated hyperlipidemia and modulates BAs metabolism. Mechanically, probiotics reduced the inflammatory factors and increased the gut barrier. In addition, *Parabacteroides distasonis* has been reported to alleviate hepatic steatosis via increasing alternative BA synthesis pathway (Kuang et al., 2023). Prebiotics are a group of nutrients in the diet that are resistant to digestion but can be fermented by the intestinal microflora, including fructo-oligosaccharides (FOS) and inulin. The fermentation of prebiotics by gut microbiota produces short-chain fatty acids (SCFAs) and affects BAs, though this is not well-documented. SCFAs may directly regulate BA synthesis and alter the microbial composition in the gut, which in turn can modify the profile of BAs. Currently, FMT is increasingly supported by evidence as a viable therapy option for NAFLD. In contrast to probiotics, FMT offers a broad spectrum of healthy bacteria that help to reshape the gut microbial dysbiosis. It is well established that FMT from lean donors enhances insulin sensitivity in individuals with metabolic syndrome (Vrieze et al., 2012). Xue et al. performed a randomized clinical trial and discovered that FMT attenuated fatty liver disease by facilitating intestinal microflora reconstruction (Xue et al., 2022). The long-term effects and potential side effects of FMT for NAFLD require further investigation. Potential risks include changes in the recipient’s gut microbial diversity that could lead to unforeseen health issues, including gastrointestinal infections, or exacerbation of other inflammatory conditions. Moreover, the durability of the treatment’s effectiveness remains uncertain.

### 5.3 Metabolic surgery as a therapeutic option for NAFLD

Metabolic surgery commonly includes vertical sleeve gastrectomy (VSG) and Roux-en-Y gastric bypass (RYGB). Numerous clinical studies and animal experiments have provided evidence that the histopathology of NAFLD improves significantly after metabolic surgery, with this improvement being closely linked to weight loss (Aguilar-Olivos et al., 2016; Axelrod et al., 2023). However, changes in BAs and the gut microbiota also play a pivotal role in ameliorating NAFLD (Pérez-Rubio et al., 2023). Both human and animal studies have shown that following RYGB surgery, there is a significant increase in both fasting and postprandial BA levels, a marked rise in the ratio of 12α-OH/non-12α-OH BAs, and a reversal in the primary to secondary BA ratio. Additionally, levels of related factors such as FGF19 and GLP-1 are also significantly elevated (Ahmad et al., 2013; Bhutta et al., 2015; Dutia et al., 2016; Lalloyer et al., 2023). These alterations in BA levels do not manifest immediately post-surgery but exert a prolonged influence. Some studies suggest that the mechanisms underlying RYGB’s alleviation of type 2 diabetes may be linked to the increase in FGF19, upregulation of CYP7A1 gene expression, and an overall rise in bile acids (Gerhard et al., 2013). Research shows that cholesterol is converted into bile acids and excreted, with the alternative BA synthesis pathway enhanced due to significant increases in CYP27A1 and CYP7B1 expression levels. This could be a mechanism through which RYGB improves NAFLD by regulating hepatic and systemic cholesterol as well as BA metabolism (Lalloyer et al., 2023). Interestingly, bile diversion surgery in diet-induced obese mice has shown that diverting bile to the ileum induces physiological changes similar to those seen with RYGB, including significant weight loss, improved glucose tolerance, and sustained improvement in liver fat deposition, with a notable increase in bile acids, especially conjugated T-β-MCA (Flynn et al., 2015). Moreover, the gut-brain axis might be involved in the metabolic surgery. BAs in serum and brain are gradually elevated after metabolic surgery, which attenuates cocaine-induced elevations in accumbal dopamine via TGR5 pathway (Reddy et al., 2018). Neuron-specific TGR5 activation exhibits anorexigenic actions via Rho-ROCK-action pathway, thus decreasing neuropeptide Y secretion (Perino et al., 2021). *In vivo*, deletion of TGR5 significantly increases food intake. Dysregulation of dopamine signaling can lead to overeating, especially of high-fat or high-sugar foods, contributing to the development of NAFLD. The specific mechanisms by which the gut-brain axis mediates the metabolic surgery effects require further investigation. While metabolic surgery can provide significant health benefits for NAFLD, it is essential to consider the potential disadvantages, including postoperative complications, nutritional deficiencies, and irreversible alterations to the digestive system (Kheirvari et al., 2020).

## 6 Conclusions and perspectives

Dysregulated BA signaling has been implicated in the development and progression of NAFLD. Therefore, understanding how BAs influence host metabolism is crucial for developing strategies to manipulate BA levels effectively in treating the disease. Although the regulation of the BA signaling holds promising therapeutic potential for NAFLD, there are currently no BA analogs or drugs targeting BA signaling available for NAFLD treatment. The principal challenge lies in developing a tissue-specific drug or non-absorbed drug that regulates BA signaling, thereby improving the metabolic dysfunction in NAFLD without causing significant adverse effects. Therefore, there is an urgent need for further research to explore the relevant molecular mechanisms of BAs in the progression of NAFLD and to develop novel drugs that can be applicated early in the treatment of NALFD. In addition, reshaping the gut microbiota to gently modulate BA signaling is also an important strategy for NAFLD treatment. Notably, FMT has emerged as a safe and efficient therapeutic approach for NAFLD. Furthermore, combination therapy, integrating multiple bile acid-targeted therapies, may enhance treatment efficacy and patient outcomes. However, there are several limitations in the current approaches to targeting BAs for NAFLD treatment. First, the intricate role of BAs in liver metabolism and systemic health remains inadequately understood, potentially leading to unintended off-target effects and systemic toxicity. Second, there are significant concerns regarding the long-term safety of modifying BA pathways, particularly because high doses of BAs may act as pro-carcinogenic agents. Third, the necessity for more phase 2 and 3 clinical trials to evaluate their long-term efficacy and safety is critical. Taken together, dysregulated BA signaling has been demonstrated to be involved in the development and progression of NAFLD. Thus, understanding the mechanism by which how BAs impact the host metabolism can help us in deciding how to manipulate the levels of BAs for treat the disease.

## Author contributions

MW: Writing – review & editing, Writing – original draft, Supervision, Investigation, Funding acquisition, Conceptualization. WT: Writing – review & editing. GH: Writing – review & editing, Supervision.

## Glossary

ASBT: Apical sodium-dependent bile acid transporter
BA: Bile acid
BAs: Bile acids
BAT: Brown adipose tissue
BSH: Bile salt hydrolase
BSEP: Bile salt export pump
WAT: White adipose tissue
CA: Cholic acid
CDCA: Chenodeoxycholic acid
ChREBP: Carbohydrate response element binding proteins
CYP7A1: Cholesterol 7-alpha hydroxylase
CYP8B1: Sterol 12α-hydroxylase
CYP27A1: Sterol-27α-hydroxylase
CYP7B1: Oxysterol 7α-hydroxylase
DCA: Deoxycholic acid
DIO2: Type II iodothyronine deionidinase
ERK1/2: Extracellular regulated protein kinases
FGF15/19: Fibroblast growth factor 15/19
FGFR4: Fibroblast growth factor receptor 4
FXR: Farnesoid X receptor
FOS: Fructo-oligosaccharides
FMT: Fecal microbiota transplantation
GLP-1: Glucagon-like peptide-1
GLP-2: Glucagon-like peptide-2
Gly-MCA: Glycine-β-muricholic acid
GLUT4: Glucose transporter 4
G6Pase: Glucose 6-phosphatase
HFD: High-fat diet
HCC: Hepatocellular carcinoma
HDCA: Hyodeoxycholic acid
HCA: Hyocholic acid
LCA: Lithocholic acid
LXR: Liver X receptor
LRH-1: Liver receptor homolog-1
LPS: Lipopolysaccharide
MCAs/α/β-MCA: Muricholic acids/α/β-muricholic acid
MRP2: Multidrug resistance-associated protein 2
NAFLD: Nonalcoholic fatty liver disease
NAFL: Non-alcoholic fatty liver
NASH: Nonalcoholic steatohepatitis
NTCP: Sodium dependent taurocholate co-transporting polypeptide
OSTα/β: Organic solute transporter subunit α and β
OCA: Obeticholic acid
PYY: Peptide YY
PXR: Pregnane X receptor
PEPCK: Phosphoenolpyruvate carboxykinase
RYGB: Roux-en-Y gastric bypass
SHP: Small heterodimer partner
SREBP-1c: Steroid response element binding protein-1c
SCFAs: Short-chain fatty acids
SIPR2: Sphingosine-1-phosphate receptor 2
TGR5: G protein-coupled bile acid receptor
UCP1: Uncoupling protein1
UDCA: Ursodeoxycholic acid
VDR: Vitamin D receptor
