# Supplementary material for: Regulating bile acids signaling for NAFLD: molecular insights and novel therapeutic interventions
Source: Front Microbiol. 2024 Jun 3;15:1341938. doi: 10.3389/fmicb.2024.1341938 (PMC11180741; doi:10.3389/fmicb.2024.1341938)
Supplement: Supplementary file 1 [file Table_1.docx]

| **Receptor** | **Expression** | **Ligands** | **Key functions in NAFLD** | **Therapeutic Implications** |
| --- | --- | --- | --- | --- |
| FXR (Farnesoid X Receptor) | white adipose tissue, adrenal glands, kidneys, and immune cells | CDCA > DCA > CA > LCA | Regulates bile acid synthesis, lipid metabolism, and glucose homeostasis; anti-inflammatory effects | FXR agonists like obeticholic acid are being investigated for their potential to treat NAFLD and NASH |
| TGR5 (G Protein-Coupled Bile Acid Receptor) | brown adipose tissue (BAT), enteroendocrine L cells, white adipose tissue (WAT), gallbladder, skeletal muscle, islet α and β cells, immune cells, astrocytes and neurons | LCA>DCA>CDCA>CA | Modulates glucose metabolism, energy expenditure; anti-inflammatory effects in macrophages | Research on TGR5 agonists is exploring their utility in metabolic disorders including NAFLD |
| VDR (Vitamin D Receptor) | Liver, intestine, pancreas, immune cells | LCA, 3-keto-LCA | inhibits BA synthesis via the FGF19-CYP7A1 pathway, and regulates immune function, inflammation, and cell proliferation | Vitamin D supplementation and analogs are under study for their effects on liver health and inflammation in NAFLD |
| PXR (Pregnane X Receptor) | Liver, intestine | 3-keto-LCA, LCA, CDCA, DCA, CA | Regulates xenobiotic metabolism; modulates bile acid detoxification and inflammatory responses | Potential therapeutic targeting in NAFLD to modulate liver detoxification and reduce inflammation |
| SIPR2(sphingosine-1-phosphate receptor 2) | Liver, immune Cells, skeletal System | Conjugated BAs (TCA, GCA, GDCA, TDCA, TUDCA) | regulates cell proliferation, immunity, inflammation, apoptosis | Involved in lipid and glucose metabolism |

Supplementary Table S1. BA receptors and their functions in NAFLD
